# Determining the clinicopathological significance of the VI-RADS ≧4 group: a retrospective study

**DOI:** 10.1186/s12894-024-01452-5

**Published:** 2024-03-20

**Authors:** Shunsuke Ikuma, Jun Akatsuka, Hayato Takeda, Yuki Endo, Tomonari Kiriyama, Tsutomu Hamasaki, Go Kimura, Yukihiro Kondo

**Affiliations:** 1 Department of Urology, Nippon Medical School Hospital, 1-1-5 Sendagi, Bunkyo-Ku, Tokyo, 113-8603 Japan; 2 Department of Radiology, Nippon Medical School Hospital, 1-1-5 Sendagi, Bunkyo-Ku, Tokyo, 113-8603 Japan

**Keywords:** Bladder cancer, Multiparameter magnetic resonance imaging, Reporting and data system, Scoring

## Abstract

**Background:**

The Vesical Imaging Reporting and Data System (VI-RADS) is widely used for predicting muscle-invasive bladder cancer (MIBC). This study aimed to determine the clinicopathological significance of the VI-RADS ≧4 (VI≧4) group.

**Methods:**

Patients who underwent transurethral resections of bladder tumors during the study period and preoperative magnetic resonance imaging were considered. The patients were pathologically diagnosed with urothelial carcinoma (UC). We first compared the results of patients with VI-RADS scores of 3 and 4 to determine the cut-off score for MIBC; thereafter, the patients were divided into the VI≧4 and VI-RADS ≦3 (VI≦3) groups using VI-RADS. The clinicopathological significance of the VI≧4 group was examined retrospectively by comparing the characteristics of each group.

**Results:**

In total, 121 cases were examined, of which 28 were pathologically diagnosed with MIBC. Of the 28 MIBC cases, three (10.7%) had a VI-RADS score of ≦3, and 25 (89.3%) had a VI-RADS score of ≧4. Of the 93 NMIBC cases, 86 (92.5%) had a VI-RADS score of ≦3, and seven (7.5%) had a VI-RADS score of ≧4. The diagnostic performance of the VI-RADS with a cut-off score of 4 was 89.3% for sensitivity, 92.5% for specificity, and an area under the curve (AUC) of 0.91. Contrastingly, for a cut-off score of 3, the sensitivity was 89.3%, specificity was 62.0%, and AUC was 0.72. A VI-RADS score of ≥ 4 could predict MIBC. In the VI≧4 group, 30 of 32 (93.8%) patients had high-grade tumors. The VI≧4 group had significantly more high-grade bladder cancers than the VI≦3 group (*p* < 0.001 OR = 31.77 95%CI:8.47–1119.07). In addition, the VI≧4 group had more tumor necrosis (VI≧4 vs VI≦3, *p* < 0.001 OR = 7.46 95%CI:2.61–21.34) and more UC variant cases (VI≧4 vs VI≦3, *p* = 0.034 OR = 3.28 95%CI:1.05–10.25) than the VI≦3 group.

**Conclusions:**

This study suggests that VI-RADS has a high diagnostic performance in predicting MIBC and that VI-RADS could diagnose high-grade tumors, necrosis, and UC variants.

## Background

Bladder cancer is the sixth most common cancer in men, with an estimated 573,278 new cases and 212,536 deaths worldwide in 2020 [1]. Treatment strategies differ between non-muscle-invasive bladder cancer (NMIBC) and muscle-invasive bladder cancer (MIBC). Therefore, an accurate diagnosis of muscle invasion in bladder cancer is important in the treatment strategy for bladder cancer.

The Vesical Imaging Reporting and Data System (VI-RADS) has become a common method for predicting MIBC. Recent reports have demonstrated the high diagnostic performance of VI-RADS [2]. However, it is unclear whether a VI-RADS score of 3 or 4 is a better cut-off score for predicting MIBC. More importantly, the clinicopathological diagnostic value of the VI-RADS, other than in predicting muscle invasion of bladder cancer, remains unknown.

This study determined the clinicopathological significance of the VI≧4 group. By identifying the clinicopathological characteristics of the VI≧4 group, we aimed to predict the pathological characteristics and presence of MIBC using VI-RADS assessment.

## Methods

### Patient population

**Fig. 1:**
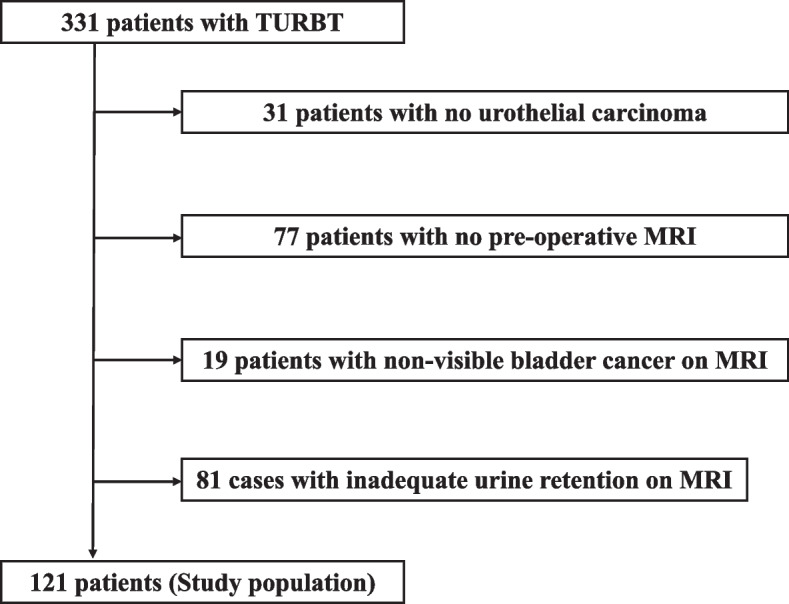
Flow chart of study population selection

From January 2019 to May 2021, 329 patients with suspected bladder cancer based on clinical findings, cystoscopy, computed tomography (CT), and magnetic resonance imaging (MRI) underwent transurethral resection of bladder tumor (TURBT) at the Nippon Medical School Hospital. As shown in Fig. 1, we excluded 31 patients who did not have pathological urothelial carcinoma (UC), 77 patients who did not undergo preoperative MRI, 19 patients with non-visible bladder cancer on MRI (with an average tumor diameter of 6.5 mm), and 81 cases in which the diagnosis of non-visible bladder cancer was due to inadequate urine retention (with an average urine volume of 36.4 mL). The 19 patients with bladder cancer were not visible on MRI due to the small tumor size, and the point of contact between the tumor and the bladder wall could not be seen. Ultimately, 121 patients were included in this study.

### Imaging analysis

MRI examinations were performed using 3.0-T equipment (SIGNA Architect, GE Healthcare, Waukesha, WI) or 1.5-T equipment (MAGNETOM Avanto Aera or Sola, Simens Healthcare, Erlangen, Germany).

The protocols for T2-weighted imaging (T2WI), diffusion-weighted imaging (DWI), and dynamic contrast-enhanced (DCE) MRI were based on technical considerations for image acquisition described in the VI-RADS report by Panebianco et al. [3].

The VI-RADS scoring was performed retrospectively and independently by two urologists. The readers were blinded to the clinical, surgical, and histopathological results associated with the images. Each reader independently evaluated the images. The final five-point VI-RADS total score was compiled using individual T2WI, DWI, and DCE-MRI scores according to the VI-RADS guidelines [3].

The VI-RADS scores were defined as follows:

- **VI-RADS score 1:** Uninterrupted low signal intensity (SI) line representing muscularis integrity (size, < 1.0 cm).
- **VI-RADS score 2:** Similar to VI-RADS score 1, except for a size > 1.0 cm and a thickened inner layer.
- **VI-RADS score 3:** Disappearance of category 2 findings, but no clear disruption of low SI muscularis layer.
- **VI-RADS score 4:** Interruption of low SI line suggesting extension into the muscularis layer.
- **VI-RADS score 5:** Extension of the intermediate SI tumor to extravesical fat.

**Fig. 2:**
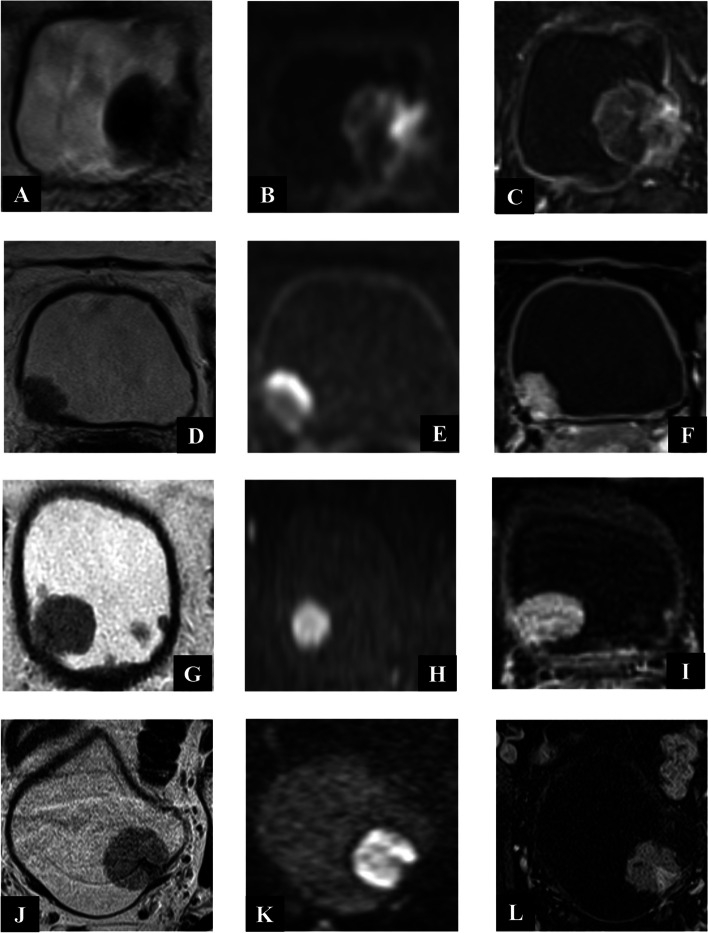
Examples of VI-RADS scores in our cases. VI-RADS 5 lesion on mp-MRI. **A** T2WI (**B**) DWI (**C**) DCE. VI-RADS 4 lesion on mp-MRI. **D** T2WI (E)DWI (**F**) DCE. VI-RADS 3 lesion on mp-MRI. **G** T2WI **H** DWI (**I**) DCE. VI-RADS 2 lesion on mp-MRI. **J** T2WI (**K**) DWI (L)DCE. Abbreviations: Vesical Imaging Reporting and Data System (VI-RADS), Multiparametric Magnetic resonance imaging (mp-MRI), T2-weighted imaging (T2WI), Diffusion-weighted imaging (DWI), Dynamic contrast-enhanced (DCE)

The schematic representation and scoring of VI-RADS (mp-MRI) are as follows:** (**Fig. 2).

- **VI-RADS 1: **(muscle invasion is highly unlikely) structural category (SC), DCE, and DWI category 1.
- **VI-RADS 2: **(muscle invasion is unlikely) SC, DCE, and DWI category 2; both DCE and DWI category 2 with SC category 3.
- **VI-RADS 3: **(the presence of muscle invasion is equivocal) SC, DCE, and DWI category 3; SC category 3, DCE or DWI category 3, and the remaining sequence category 2.
- **VI-RADS 4: **(muscle invasion is likely) at least SC and/or DWI and DCE category 4; the remaining category 3 or 4; SC category 3 plus DWI and/or DCE category 4; SC category 5 plus DWI and/or DCE category 4.
- **VI-RADS 5: **(invasion of muscle and beyond the bladder is very likely) at least SC plus DWI and/or DCE category 5; the remaining category 4 or 5.

In cases without DCE-MRI, the final VI-RADS score was based on DWI. This method for assessing VI-RADS in the bi-parametric MRI (bp-MRI) was based on the method of Noh et al. [4].

The schematic representation and scoring of VI-RADS (bp-MRI) are as follows:

- **VI-RADS 1: **(muscle invasion is highly unlikely) SC and DWI category1.
- **VI-RADS 2: **(muscle invasion is unlikely) SC and DWI category 2; and SC 3 with DWI category 2.
- **VI-RADS 3: **(presence of muscle invasion is equivocal) SC and DWI category 3.
- **VI-RADS 4: **(muscle invasion is likely) at least SC and/or DWI category 4; SC category 3 with DWI category 4; and SC category 5 with DWI category 4.
- **VI-RADS 5: **(invasion of muscle and beyond the bladder is very likely) SC and DWI category 5.

### Standard of reference

TURBT was performed on all 121 patients. Our method of tumor resection with TURBT involved resectioning all lesions of the suspected tumor and two additional deep layers in the index tumor area. Resected specimens were preserved in 10% formalin for pathological diagnosis. The specimens were evaluated for the presence of muscle invasion by a dedicated uropathologist with 20 years of experience and blinded to MRI during evaluation.

### Statistical analysis

Statistical analysis was performed using SPSS version 29 (SPSS Inc, Chicago, Illinois). *P* < 0.05 was considered statistically significant. We used receiver operating characteristic (ROC) curve analysis and the area under the ROC curve (AUC) to assess the diagnostic performance of the VI-RADS score, and we also evaluated the predictive ability of the VI-RADS score for MIBC by calculating the sensitivity, specificity, accuracy, positive predictive value, and negative predictive value. The weighted kappa coefficient was used to evaluate the inter-reader agreement.

We first compared the results of patients with VI-RADS scores of 3 and 4 at our institution to define the cut-off score for MIBC. Based on this cut-off value, we divided the 121 cases into two groups: VI-RADS≧4 (VI≧4) and VI-RADS≦3 (VI≦3), using VI-RADS evaluation. We retrospectively examined the pathological significance of VI≧4 by comparing the clinicopathological characteristics of each group using results from readers with high diagnostic performance.

Continuous variables were compared using either the Mann–Whitney U test or Spearman's correlation coefficient, while categorical variables were analyzed using Chi-square analysis.

## Results

Table 1 shows the characteristics of this study. The mean age of the patients was 72.7 years (range: 32–98 years, median: 73.0 years). Of the 121 patients, 103 (85.1%) were males, and 18 (14.9%) were females. Based on the MRI findings, the mean tumor diameter was 19.8 mm (range: 3–59 mm), with a median diameter of 16.0 mm. Further, 98 patients (81.0%) had tumors < 3 cm in diameter, and 23 patients (19.0%) had tumors ≥ 3 cm; 100 patients (82.6%) had a single tumor, and 21 patients (17.4%) had multiple tumors. Preoperative urine cytology was classified according to The 2015 Japan Reporting System for Urinary Cytology [5]. Preoperative urine cytology showed that 86 tumors ( 71.1%) were classified as atypical cells, class III or lower, while 35 (28.9%) were suspected to be malignant, class IV or higher. The histopathological findings showed that all 121 patients had UC; 93 patients (76.9%) had NMIBC, and 28 (23.1%) had MIBC. In this study, a second TURBT was performed on 54 out of 121 patients (44.6%). Two patients (3.7%) were upstaged from pTa to pT1, but no cases of NMIBC were upstaged to MIBC. The muscle layer was sampled in 116 out of 121 cases (98.3%); the remaining five cases without muscle layer sampling underwent second TURBT, which included muscle layer sampling. All pathology results considered the outcomes of the second TURBT.

**Table 1:** Patients’ characteristics

Variables	Total(n)		121	
Age (yr)	mean	72.7	Median	73.0
Sex (n)	Male	103	Female	18
Tumor size (mm)	mean	19.8	Median	16.0
Tumor size	< 3 cm	98	≧3 cm	23
Number of tumors	Single	100	Multiple	21
Urine cytology (n)	< Class4	86	≧Class4	35
NMIBC/MIBC	NMIBC	93	MIBC	28
Histology grade (n)	Low (G1 or G2)	51	High (G3)	70
CIS (n)	( +)	15	(-)	106
Tumor necrosis (n)	( +)	19	(-)	102
UC Variant (n)	( +)	16	(-)	107

*Abbreviations*: *MIBC* Muscle-invasive bladder cancer, *NMIBC* Non-muscle-invasive bladder cancer, *UC* Urothelial carcinoma, *CIS* carcinoma in situ

Further, 71 cases (58.7%) had pTa, 24 (19.8%) had T1, and 26 (21.5%) ≥ T2; 70 cases (57.9%) had high-grade tumors (G3), and 51 cases (42.1%) had low-grade tumors (G1, G2). In addition, 15 patients (12.4%) had carcinoma in situ, 19 (15.7%) had tumor necrosis, and 16 (11.6%) had UC variant.

Out of the 121 patients, 28 were diagnosed with MIBC through histopathological examination. Among the 28 MIBC cases, four had a VI-RADS score of ≦3, while 24 had a VI-RADS score of ≧4 to Reader1. For Reader2, three cases had a VI-RADS score of ≦3, and 25 had a VI-RADS score of ≧4. In the NMIBC group of 93 patients, 80 had a VI-RADS score of ≦3, and 13 had a VI-RADS score of ≧4, as assessed by Reader1. Reader2 identified 86 cases with a VI-RADS score of ≦3 and seven cases with a VI-RADS score of ≧4, as detailed in Table 2. The performance of the VI-RADS assessment in detecting MIBC using VI-RADS scores 3 and 4 as the cut-offs for muscle invasion detection is shown in detail in Table 3. Using a VI-RADS cut-off of 4, Reader1 achieved an AUC of 0.86, with 85.7% sensitivity, 86.0% specificity, 91.7% accuracy, 64.9% positive predictive value, and 95.2% negative predictive value. For Reader2, the AUC was 0.91, with 89.3% sensitivity, 92.5% specificity, 86.0% accuracy, 78.1% positive predictive value, and 96.6% negative predictive value. Using a VI-RADS cut-off of 3, Reader1 achieved an AUC of 0.76, with 89.3% sensitivity, 63.4% specificity, 69.4% accuracy, 42.4% positive predictive value, and 95.2% negative predictive value. Reader2, on the other hand, obtained an AUC of 0.72, with 89.3% sensitivity, 53.8% specificity, 62.0% accuracy, 36.8% positive predictive value, and 94.3% negative predictive value. Notably, Reader2 demonstrated superior diagnostic performance compared to Reader1. For the analysis of clinicopathological features in this study, the VI-RADS scores assigned by Reader2 were used. The agreement between the two readers was substantial, with a kappa statistic of 0.78 (95%CI:0.70–0.86) for T2WI score, 0.78 (95%CI:0.70–0.85) for DWI score, 0.83 (95%CI:0.74–0.92) for DCE score, and 0.82 (95%CI:0.56–0.80) for VI-RADS score. There was excellent agreement between the readers in the assessment of VI-RADS scores (Table 4).

**Table 2:** Cases scored by VI-RADS

VI-RADS score reader1	Muscle invasion		VI-RADS score reader2	Muscle invasion	
	positive	negative		positive	negative
1	1(3.6)	18(19.4)	1	1(3.6)	18(19.4)
2	2(7.1)	41(44.1)	2	2(7.1)	32(34.4)
3	1(3.6)	21(22.6)	3	0(0)	36(38.7)
4	4(14.3)	8(8.6)	4	5(17.9)	6(6.5)
5	20(71.4)	5(5.4)	5	20(71.4)	1(1.1)

**Table 3:** Comparison of scores using 3 and 4 as cut-off scores

	VI-RADS Cut-off value	SSY(%)	SPY(%)	ACC(%)	PPV(%)	NPV(%)	AUC (95%CI)
Reader1	4	85.7	86.0	91.7	64.9	95.2	0.859( 0.773–0.944)
	3	89.3	63.4	69.4	42.4	95.2	0.764( 0.671–0.857)
Reader2	4	89.3	92.5	86.0	78.1	96.6	0.909( 0.835–0.982)
	3	89.3	53.8	62.0	36.8	94.3	0.715( 0.616–0.814)

**Table 4:** Agreement between readers according to the VI-RADS score results

	Agreement value (95% CI)
	Reader1 and Reader 2
T2WI score	0.780( 0.703–0.857)
DWI score	0.777( 0.700–0.854)
DCE score	0.826( 0.737–0.915)
VI-RADS score	0.822( 0.555–0.798)

*Abbreviations*: *SSY* Sensitivity, *SPY* Specificity, *ACC* Accuracy, *AUC* Area under the receiver operating characteristic curve, *PPV* Positive predictive value, *NPV* Negative predictive value, *95%CI* 95% Confidence Interval, *T2WI* T2-weighted imaging, *DWI* Diffusion-weighted imaging, *DCE* Dynamic contrast-enhanced

We compared tumor diameters between the VI≧4 and VI≦3 groups, as assessed by Reader2, as shown in Table 5. The mean tumor diameter was 31.8 mm in the VI≧4 group (range: 10—59 mm, median: 30 mm) and 15.5 mm in the VI≦3 group (range: 3—46 mm, median: 13 mm). Univariate analysis was performed for tumor diameters in the VI≧4 and VI≦3 groups. The VI≧4 group had a significantly larger mean tumor diameter than the VI≦3 group (VI≧4 vs VI≦3, *p* < 0.0001, 95%CI:0.42–0.68). In relation to tumor diameter, we also compared the two groups using a cut-off value of 3 cm for tumor size. Sixteen cases (50.0%) in the VI≧4 group and seven cases (7.9%) in the VI≦3 group had a tumor diameter of ≧3 cm. The VI≧4 group had significantly more tumors with a diameter ≧3 cm than the VI≦3 group (VI≧4 vs VI≦3 *p* < 0.0001, OR = 31.77, 95%CI:8.47–119.07).

**Table 5:** Comparison of clinicopathological features of VI-RADS≧4 and ≦3 by Reader2

Variable	Total (*n* = 121)	VI-RADS≧4 (*n *= 32)	VI-RADS≦3 (*n* = 89)	VI-RADS≧4 vs VI-RADS≦3	
				Univariate analysis	
				*P* = value	OR (95%CI)
Mean age (yr)	72.7	75.6	71.7	0.145	(-0.09–0.27)
Sex (n)					
Male	103	24	79	0.166	2.04
Female	18	8	10		(0.73–5.68)
Tumor size (n)					
Tumor mean size (mm)	19.8	31.8	15.5	< 0.001	(0.42–0.68)
< 3 cm	98	16	82	< 0.001	31.77
≧3 cm	23	16	7		(8.47–119.07)
Number of tumors (n)					
Single	100	29	71	0.165	0.41
Multiple	21	3	18		(0.11–1.49)
Urine cytology (n)					
≦ ClassIII	86	18	68	0.031	2.52
≧ ClassIV	35	14	21		(1.07–5.91)
Histology grade (n)					
Low (G1 or G2)	51	2	49	< 0.001	18.38
High (G3)	70	30	40		(4.14–81.62)
CIS (n)					
( +)	15	4	11	0.984	1.01
( −)	106	28	78		(0.30–3.44)
Tumor necrosis (n)					
( +)	19	12	7	< 0.001	7.46
( −)	102	20	82		(2.61–21.34)
UC Variant (n)					
( +)	16	8	8	0.022	3.38
( −)	105	24	81		(1.15–9.95)

*Abbreviations*: *UC* Urothelial carcinoma, *CIS* Carcinoma in situ

We also compared preoperative urine cytology between the VI≧4 and VI≦3 groups. As shown in Table 5, 14 out of 32 (43.8%) patients in the VI≧4 group had urine cytology class≧IV. In contrast, 21 out of 89 (23.6%) patients in the VI≦3 group had urine cytology class≧IV. We compared the percentage of urine cytology class ≧IV in each group (VI≧4 vs VI≦3, *p* = 0.031, OR = 2.52, 95%CI:1.07–5.91). The VI≧4 group had significantly more cases with urine cytology class ≧IV than the VI≦3 group.

Furthermore, as shown in Table 3, 30 out of 32 (93.8%) patients in the VI≧4 group had high-grade tumors. In contrast, 40 out of 89 (44.9%) patients in the VI≦3 group had high-grade tumors. We compared the percentage of high-grade tumors in each group (VI≧4 vs VI≦3, *p*<0.001, OR=18.38, 95%CI:4.14-81.62). The VI≧4 group had significantly more high-grade tumors than the VI≦3 group.

Tumor necrosis was observed in 12 of the 32 patients (37.5%) in the VI≧4 group, while the VI≦3 group had tumor necrosis in seven out of 89 cases (7.9%). Univariate analysis was performed for tumor necrosis in the VI≧4 and VI≦3 groups (VI≧4 vs VI≦3, *p* < 0.001, OR = 7.46, 95%CI:2.61–21.34). The VI≧4 group had significantly more cases of tumor necrosis in bladder cancer than the VI≦3 group.

In this study, UC variants were found in 16 out of 121 cases, with five cases featuring multiple UC variants within the same tumor. The types of UC variants included lipid cell variants in 12 cases, plasmacytoid variants in two cases, microcystic variants in four cases, and micropapillary variants in four cases. There were eight cases of UC variants in the VI≧4 group and eight cases in the VI≦3 group. Univariate analyses were performed for patients with bladder cancer and UC variants in each group (VI≧4 vs VI≦3, *p* = 0.022, OR = 3.38, 95%CI:1.15–9.95). The VI≧4 group had significantly more cases of UC variants in bladder cancer than the VI≦3 group.

Other comparisons were performed for sex and the number of tumors, but no significant differences were noted between the VI≧4 and VI≦3 groups.

## Discussion

The diagnosis of muscle layer invasion in bladder cancer is of immense significance due to its direct impact on treatment decisions and prognostic outcomes. Local staging of bladder cancer is primarily based on pathology specimens obtained by TURBT. In some cases, a second TURBT is recommended when there is a T1 high-grade or when no muscular layer sample is present in the specimen at the first TURBT. Initial TURBT is sometimes inadequate, with residual tumors reported in 33%–76% of cases and up-staging in 0%–32% of cases [6]. In this study, second TURBT was performed in 54 out of 121 cases (44.6%). Residual tumor was discovered in 22 cases (39.3%), and up-staging occurred in two cases (3.7%). A second TURBT is effective for resection of residual tumor and obtaining a muscular layer sample but is not without the risk of bleeding and perforation. To mitigate the risk of potential complications of TURBT, imaging tools are recommended. CT can detect tumor invasion outside the bladder, but it cannot provide detailed images of the border between the tumor and the muscle layer. Therefore, CT cannot be used for local diagnosis of bladder cancer.

Since the VI-RADS was first reported in 2018, it has become a popular diagnostic method for predicting muscle invasion in bladder cancer. Several meta-analyses of the diagnostic performance of the VI-RADS have been reported [2]. In 2020, Woo et al. reported a high diagnostic performance of the VI-RADS with 83% sensitivity, 90% specificity, and the area under the hierarchical summary receiver operating characteristic (HSROC) curve was 0.94 [6].

VI-RADS is a system that predicts muscle layer invasion, with its scores reflecting the extent of invasion. In the study by Panebianco et al., no cut-off score was set [3]. The present study examined the diagnostic performance of a cut-off VI-RADS score as a predictive tool for MIBC and found the use of a cut-off score to enhance the usefulness of the VI-RADS in clinical practice.

In 2022, Del Giudice et al. reported a high diagnostic performance in their meta-analysis of the VI-RADS score; with a cut-off value of 3, it showed a sensitivity of 87%, a specificity of 86%, and an area under the HSROC curve of 0.93. A cut-off value of 4 showed a sensitivity of 78%, a specificity of 94%, and an area under the HSROC curve of 0.91 [2]. In comparison, this study showed a sensitivity of 89.3%, a specificity of 92.5%, a positive predictive value of 78.1%, and an AUC of 0.91. The diagnostic performance of VI-RADS in this study was slightly lower than that reported in other reports. The reasons for this are that the present study evaluated VI-RADS using 1.5 T-MRI and bp-MRI, and that the study population was smaller than that in other studies. [7]

Furthermore, in the present study, the VI≧4 group exhibited larger tumor diameters than the VI≦3 group. A large tumor diameter was identified as a preoperative factor associated with VI≧4. The relationship between tumor diameter and pathological findings is further discussed below.

Subsequently, by comparing the clinicopathological characteristics of each group, we retrospectively examined the characteristics of the VI≧4 group. This study revealed a higher prevalence of high-grade (G3) tumors in the VI≧4 group than in the VI≦3 group. The VI≧4 group was diagnostic for MIBC and suggested the possibility of predicting high-grade bladder cancer. Until recently, it was believed that low-grade bladder tumors progressed to high-grade tumors through the accumulation of genetic abnormalities [8, 9]. However, low-grade and high-grade tumors are genetically exclusive [10–12]. Therefore, low-grade tumors rarely become high-grade tumors. From this perspective, almost all cases in the VI≧4 group exhibited high-grade tumors (G3) in the results of the present study. The VI≧4 group had significantly more high-grade bladder tumors than the VI≦3 group, suggesting that the VI≧4 group could be associated with a diagnosis of high-grade tumors. Therefore, the VI≧4 cases should be considered for a treatment plan that includes total cystectomy and systemic treatment.

Regarding tumor grade, there were significantly more cases of urine cytology class≧4 in the VI≧4 group than in the VI≦3 group. Urine cytology is a pathological method used to diagnose atypia of urothelial exfoliated cells in the urine. Its sensitivity for low-grade NMIBC is low, while it is high for high-grade urothelial carcinoma [13]. In this study, we observed a higher prevalence of urine cytology class ≧4 in the VI≧4 group, which contains more high-grade tumors.

In relation to the tumor grade, there were significantly more cases of tumor necrosis in the VI≧4 group than in the VI≦3 group. In this case, the mean diameter of the MRI of the VI≧4 group was 31.8 mm (range: 10—59 mm, median: 30 mm). It was significantly larger than that of the VI≦3　group. Armin et al. suggested that tumors > 3 cm in diameter are high-grade tumors with necrosis [14]. This study suggests that patients in the VI≧4 group may be diagnosed with high-grade UC based on tumor size and necrosis.

The major pathological type of bladder cancer is UC; non-UC is considered rare, accounting for approximately 5% of all cases [15]. Further, urothelial bladder carcinoma has several subtypes. The presence of the UC variant is thought to be relatively rare, but recent reports have shown that it is present in approximately 20–25% of all cases [16, 17]. In this study, UC variants were identified in 16 out of 121 patients (11.6%) with high-grade tumors, and the VI≧4 group showed significantly more UC variants than the VI≦3 group.

The most common UC variant in this study was the lipid cell variant, which often occurs with high-grade UC or the micropapillary variants of UC [18]. Lopez et al. reported that the lipid cell variant is diagnosed at an advanced stage, with high grade and high mortality. [18] In this study, eight of 12 lipid cell variants cases were VI≧4, and nine of 12 cases were advanced stage (> pT2). All lipid cell variant cases were high-grade tumors, and three of 12 cases had tumor necrosis. Therefore, this study suggested that the VI≧4 group contains a considerable number of highly malignant lipid cell variants. Lopez et al. also suggest the potential presence of a lipid cell variant, which can pose diagnostic challenges. We believe that our study successfully diagnosed a latent lipid cell variant [18].

The micropapillary variant often occurs with lymph vascular invasion and is also diagnosed at an advanced stage. Kamat et al. report a 5-year overall survival rate of 54% and a 10-year survival rate of 27% for the micropapillary variant, which is a poor prognosis. To improve its prognosis, immediate total cystectomy is a viable option [19]. In this study, the micropapillary variant had lymphovascular invasion in two of four cases and VI≧4 in three of four cases. Because the VI≧4 group may include the micropapillary variant, lymph node metastasis must be carefully evaluated during diagnosis.

The microcystic variant is a relatively rare UC variant, and thus, its clinical characteristics and prognostic outcomes are controversial [20]. Lopez et al. concluded that there was no significant difference in prognosis when the microcystic variant was compared with conventional UC [21]. In this study, the microcystic variant cases were all NMIBC cases. However, three of four cases were high-grade tumors, and thus, this variant should be followed up for recurrence and metastasis.

Finally, two patients with the plasmacytoid variants were included in the VI≧4 group. The plasmacytoid variant has a poor prognosis and requires a treatment plan that includes surgery and chemotherapy [22]. Therefore, immediate treatment is recommended and possible if it can be predicted by preoperative MRI.

This study examined the possibility that the VI≧4 group may provide predictive insights on the clinicopathological features. The VI≧4 group had a higher incidence of MIBC and high-grade tumors. Furthermore, this study suggests that the VI≧4 group may have potentially high-grade bladder tumors with tumor necrosis and UC variants. The potential significance of the VI ≧4 group is that it has the added value of predicting not only MIBC but also some pathological features.

## Limitations

Our study had some limitations. First, the study was retrospective. Second, the sample size was small. Third, the study was performed using 1.5-T MRI, which has slightly less contrast resolution than 3.0-T MRI. Fourth, DCE-MRI is an essential part of the VIRADS technique. However, this study included cases with bp-MRI.

## Conclusions

In our study, the VI-RADS showed a high diagnostic performance. The VI≧4 group had a significantly higher number of high-grade tumors. The VI≧4 group also had significantly more cases of tumor necrosis and UC variants.

## Data Availability

The datasets used and/or analyzed during the current study are available from the corresponding author on reasonable request.

## Abbreviations

NMIBC: Non-muscle-invasive bladder cancer
MIBC: Muscle-invasive bladder cancer
VI≧4: VI-RADS≧4
VI-RADS: Vesical Imaging Reporting and Data System
CT: Computed tomography
MRI: Magnetic resonance imaging
TURBT: Transurethral resections of bladder tumors
UC: Urothelial carcinoma
VI≦3: VI-RADS≦3
T2WI: T2-weighted imaging
DWI: Diffusion-weighted imaging
DCE: Dynamic contrast-enhanced
SI: Signal intensity
bp-MRI: Biparametric MRI
SC: Structural category
ROC: Receiver operating characteristic
AUC: Area under the curve
HSROC: The hierarchical summary receiver operating characteristic
NCCN: The National Comprehensive Cancer Network
EAU: The European Association of Urology
